# Phonetic complexity affects children’s Mandarin tone production accuracy in disyllabic words: A perceptual study

**DOI:** 10.1371/journal.pone.0182337

**Published:** 2017-08-14

**Authors:** Puisan Wong, Winifred Strange

**Affiliations:** 1 Division of Speech and Hearing Sciences, Faculty of Education, The University of Hong Kong, Pokfulam, Hong Kong; 2 Ph.D. Program in Speech-Language-Hearing Sciences, The Graduate School and University Center of the City University of New York, New York, New York, United States of America; Nanjing University, CHINA

## Abstract

This is the first study to examine the effect of phonetic contexts on children’s lexical tone production. Mandarin tones in disyllabic words produced by forty-four 2- to 6-year-old children and twelve mothers were low-pass filtered to eliminate lexical information. Native Mandarin-speaking adults categorized the tones based on the pitch information in the filtered stimuli. All mothers’ tones were categorized with ceiling accuracy. Counter to the findings in most previous studies on children’s tone acquisition and the prevailing assumption in models of speech development that children acquire suprasegmental features much earlier than segmental features, this study found that children as old as six years of age have not mastered the production of Mandarin tones. Children’s tones were judged with significantly lower accuracy than mothers’ productions. Tone accuracy improved, while cross subject variability in tone accuracy decreased, with age. Children’s tone accuracy was affected by the articulatory complexity of phonetic contexts. Children made more errors in tone combinations with more complex fundamental frequency (F0) contours than tone sequences with simpler F0 changes. When producing disyllabic tone sequences with complex F0 contours, children tended to shift the F0 contour of the first tone to reduce the F0 change, resulting in more tone errors in the first syllable than in the second syllable and showing substantially more anticipatory coarticulation than adults. The results provide further evidence that acquisition of lexical tones is a protracted process in children. Tones produced accurately by children in one phonetic context may not be produced correctly in another phonetic context. Children demonstrate more anticipatory coarticulation in their disyllabic productions than adults, which may be attributed to children’s immature speech motor control in tone production, and is presumably a by-product of their inability to accomplish complex F0 changes within the syllable time-frame.

## Introduction

Many of the world’s languages use lexical tones to convey semantic contrasts. Studies that examined children’s acquisition of Mandarin tones yielded contradictory results; some studies reported that children acquire the four tones as early as one-and-a-half years of age [1, 2], well before they master the correct productions of vowels and consonants, while other studies reported that even five-year-old children have not mastered the production of tones [3]. Most of these studies examined children’s acquisition of monosyllabic lexical tones. No study has systematically examined children’s production of lexical tones in connected speech. Therefore, it remains unclear when children have mastered Mandarin tone productions in coarticulated speech and whether phonetic contexts have an effect on children’s tone production. This study examined whether pre-school children have mastered the production of the four Mandarin tones in coarticulated disyllabic words and whether their tone accuracy is affected by different tone combinations.

Mandarin, the most widely spoken tone language, is a syllable-timed language with predominantly disyllabic words [4]. Each syllable is a morpheme and carries one of the four full lexical tones or the neutral tone. The four full tones are equally stressed and are typically labeled as high level, rising, low dipping, and high falling, which are also referred to as Tone 1 (T1), Tone 2 (T2), Tone 3 (T3) and Tone 4 (T4), respectively. The primary and sufficient cue for the perception of Mandarin tones is the shape and height of the fundamental frequency (F0) contour [5, 6]. Intensity and duration are secondary cues, which are negligible in the presence of F0 information [5]. To achieve the percept of the four tones, four corresponding pitch targets, namely a high (H), rising (R), low (L) and falling (F) F0 contour, are reached within the time frame of the syllable [7]. Syllables produced with the same phonemic segments but different pitch targets have distinct meanings. For example, the syllable ‘ma’ produced with T1, T2, T3, and T4 means ‘mother’, ‘hemp’, ‘horse’ and ‘scold’, respectively (See [8] figure 2 for the F0 contours of the four tones produced in isolation by adults). Though the pitch target for T3 is a low F0 contour, T3 has different surface forms in different contexts. In isolation or at utterance final position, it is usually produced with a dipping-then-rising F0 contour; but in non-utterance-final position or in connected speech, it is produced with a low level or falling F0 without the final rising component. T3 also undergoes the T3 sandhi rule: when two T3s are produced as a unit such as in a disyllabic word, the first T3 is produced as T2. For this reason, the T33 (T3 followed by another T3) combination was excluded in the present study. Mandarin has a fifth tone, the neutral tone, which occurs in weakly stressed syllables in only 5–7% of the words in Mandarin [9] and was not included in this study.

Past research on the acquisition of Mandarin tone production has reported conflicting results with respect to the age and order of acquisition of Mandarin lexical tones. Earlier studies reported early acquisition of lexical tones, supporting the prevailing assumption in models of speech acquisition that supra-segmental features in speech such as prosody are acquired before segmental features of speech such as consonants and vowels [10–12]. Several studies postulated that lexical tones were acquired around the age of two years [1, 2, 13]. For example, Hua and Dodd [1] reported only two tone errors among the monosyllabic and multisyllabic productions of 129 children between the age of 1;6 and 4;6 in picture naming and picture description tasks, suggesting that children as young as 1;6 produced no tone errors [1]. A couple of studies reported that the four tones were not acquired until three years of age or later [14, 15]. For example, in Clumeck [14] two of the participants had not acquired the four tones at 2;10 and 3;5. These earlier studies elicited children’s tone productions from monosyllabic and multi-syllabic words, and from isolated productions in picture naming tasks and connected productions in picture description tasks and spontaneous speech. Children’s tone accuracy was determined by one person, who was usually the experimenter, with the support of contextual, semantic, syntactic and segmental information about the productions. Sometimes to assist transcription, the mother was asked to interpret the target form of the child (e.g., [2]). Several of the studies included only 1–4 children (e.g., [2, 13, 14]). None of the studies involved an adult reference group for comparison and the criterion for determining tone mastery was not clear (See [3, 16] for a review).

More recently, Wong and colleagues published several studies on children’s production of Mandarin tones in isolated monosyllabic words using a more controlled experimental design and reported that children as old as five years of age did not produce the four Mandarin tones in monosyllabic words with adult-like accuracy. Wong [17] and Wong [18] examined Mandarin tone productions of three-year-old monolingual children growing up in the U.S. and in Taiwan, respectively. Unlike previous studies, they employed multiple judges and controlled for tone expectation biases in tone judgments by requiring the judges to categorize children’s and adults’ tones that had been low-pass filtered to retain F0 information but eliminate lexical information. They also collected mothers’ productions and used them as a reference to compare with the children’s productions. The results showed that Mandarin-speaking children growing up in the U.S. and in Taiwan performed comparably. Adults’ filtered tone productions were identified by the judges with ceiling accuracy, whereas the four tones produced by the two groups of Mandarin-speaking children were identified with significantly lower accuracy. Among the four tones, T3, which has the most complex F0 contour, was particularly difficult for both groups of children to produce. These findings suggested that three-year-old Mandarin-learning children have not mastered the production of the four tones in isolated monosyllabic words and tones with more complex F0 patterns are more difficult for children to acquire.

Wong [19] supported the findings and validated the perceptual method adopted in Wong [17] and Wong [18] by providing detailed acoustic analysis on the tones produced by the children in Wong [17]. The pitch contours of children’s tones that were correctly identified by the judges largely matched the pitch contour of the same tones produced by adults, though not all acoustic parameters in children’s correct tones were adult-like. Children’s tones that were misidentified by the judges were acoustically different from children’s and adults’ correct productions and the acoustic parameters of the incorrect productions matched the characteristics of the tones selected by the judges.

Using the methods established in Wong et al. [17] and Wong [18, 19], Wong [3] examined the developmental course of monosyllabic Mandarin tone productions in three- to five-year-old children growing up in Taiwan and reported that even five-year-old children growing up in Taiwan did not produce any of the four tones in monosyllabic words with adult-like accuracy and the accuracy rates of the four tones followed the order of articulatory complexity of the four tones. Overall, these more recent studies suggest that children’s tone acquisition is a protracted process and children’s tone accuracy is affected by the phonetic complexity of the F0 contours. The findings also imply that the same lexical tones produced in different phonetic contexts may impose different levels of difficulty for children. To date, other than two studies that examined context effects on tone acquisition in adult second language learners of Mandarin [20] and adult heritage Mandarin speakers [21], no study has investigated the effects of phonetic contexts on children’s lexical tone acquisition, the coarticulatory patterns of children’s tone production, or the development of coarticulated lexical tones in children.

Most studies that investigated children’s development of coarticulation compared the degree of coarticulation of consonant and vowels in CV syllables between children and adults and reported contradictory and diverse results. Studies that found more anticipatory coarticulation in children’s productions proposed that young children organize their articulatory gestures by the duration of the syllable [22]. Thus, there is more overlap of gestures for different phonemes in the syllable, causing more coarticulation in children’s productions. When children grow older, their speech motor control becomes more mature, which allows them to refine their articulatory gestures and organize their articulatory gestures by phonemes, resulting in less coarticulation and more adult-like speech production [22–24]. Studies that found less coarticulation in children than adults proposed that young children start by producing speech segment by segment and do not learn to accommodate neighboring segments until they are older, resulting in less coarticulation than adults [25, 26]. Yet, other studies found similar amounts of coarticulation in children and adults [27], while still others found that the amount of coarticulation in children depended on contexts [28–30]. No study has examined tone coarticulation in children. Unlike coarticulation of consonants and vowels, which involves motor control and coordination of different articulators (e.g., lips, jaw and tongue) or different postures or movement of the tongue, coarticulation of tones employs the same articulators and mechanism–speed of vibration of the vocal folds. Examining laryngeal coarticulation in children will shed new light on factors affecting speech coarticulation in children.

**Fig 1:**
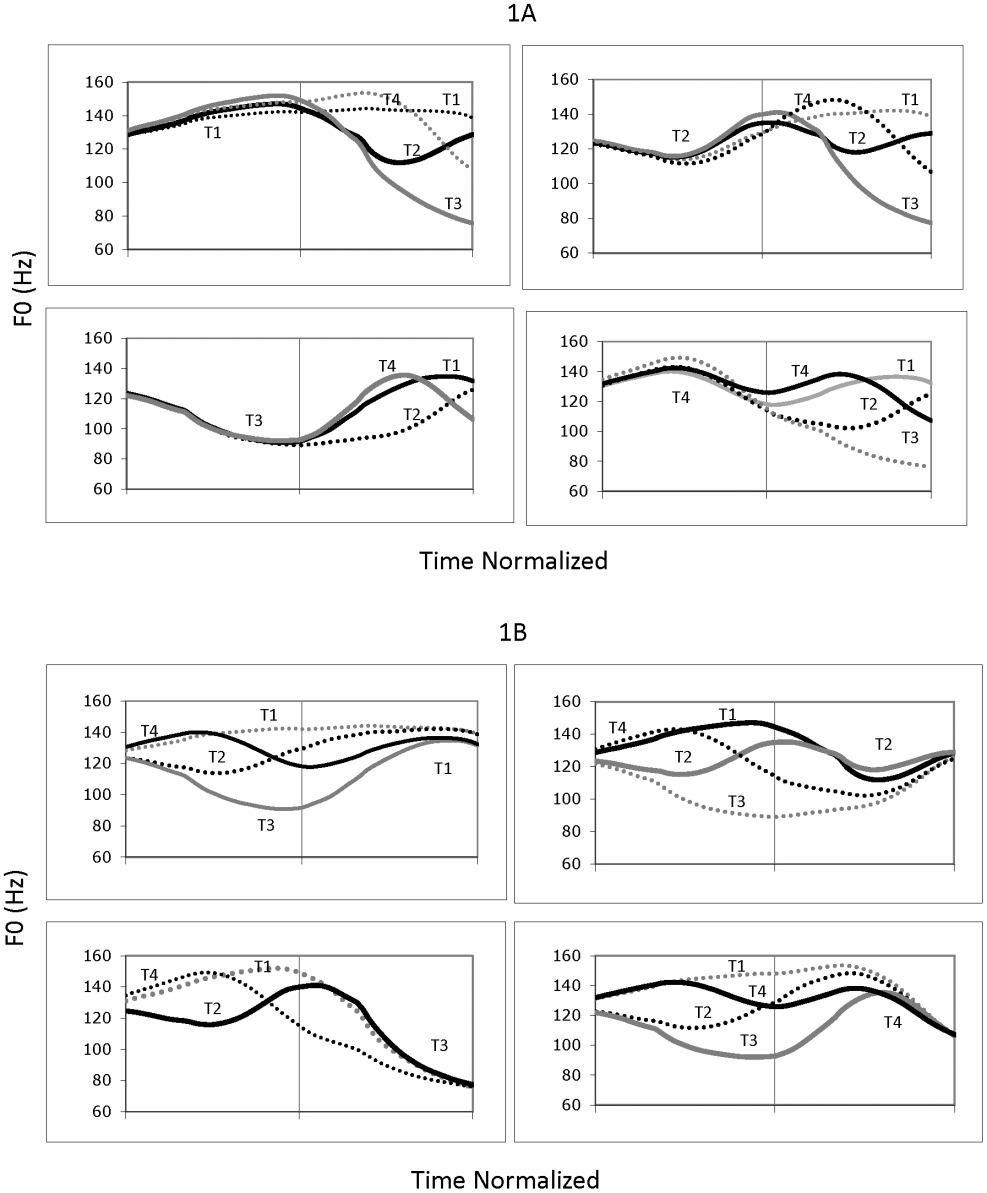
F0 contours of disyllabic mandarin tones (adapted from [31]). Mean f0 Contours of the 15 Combinations of the Four Mandarin Lexical Tones Organized by the Tones in the First Syllable. (B) Mean f0 Contours of the 15 Combinations of the Four Mandarin Tones Organized by the Tones in the Second Syllable. Note: There is no Tone 3 (Low) followed by Tone 3 (Low) combination because it is produced as Tone 2 (Rising) followed by Tone 3 (Low) due to Tone 3 sandhi rule in Mandarin. Thus, there are only 15 disyllabic lexical tone combinations for the four tones. The vertical lines represent the syllable boundaries. The contours in dotted lines represent F0 contours of compatible tone combinations whereas the contours in solid lines represent F0 contours of non-compatible tone combinations. Figures adapted from Figures 2 and 3 in “Sources of Tonal Variations in Connected Speech”, by Y. Xu, 2001, Journal of Chinese Linguistics, Monograph Series #17, P. 1–31. Courtesy of Yi Xu.

In adults’ coarticulated Mandarin tones, the F0 contour of the same tone varies depending on the syllable position and the tonal context, particularly the tone in the preceding syllable [7, 31]. For example, in isolated disyllabic words, the tones in the first syllable (S1) are less varied than the same tones in the second syllable (S2) [8, 20]. Fig 1 (adapted from [31]) shows the F0 contours of all the 15 disyllabic tone combinations (DTs) produced by adults (T33 is excluded because it is produced as T23 due to the T3 sandhi rule). Fig 1A organizes the 15 contours by the tones in S1. Fig 1B shows the same 15 disyllabic F0 contours organized by the tones in S2. Comparing the pitch contours in the left panels in Fig 1A to the pitch contours in the right panels in Fig 1B, the pitch contours of the same tone in S1 (Fig 1A left panels) display similar F0 contour shapes and small variations in pitch height, regardless of the tone in S2 (small anticipatory coarticulatory effect). However, the F0 contours of the same tone in S2 (Fig 1B right panels) are substantially different depending on the tone in S1 (large carryover coarticulatory effect). Essentially, the initial portion of S2 contains the transition from the F0 offset of S1 to the F0 onset of the target tone in S2. The tonal target (i.e., the H, R, L, and F F0 contour) for the tone in S2 is achieved only towards the end of S2 [8, 9]. Thus, overall, the F0 heights and F0 shapes of the contours of the same tones are more varied in S2 than in S1, and in about half of the cases, the contours of the tones in S2 have more complex contour shapes than the same tone in S1. It was therefore predicted that, overall, children would have more difficulties with the tones in the second syllable than the tones in the first syllable when producing disyllabic words in isolation.

From another perspective, if we consider the F0 contours of the two tones in a disyllabic word as a unit, F0 contours in some DTs are more complex than in others. Table 1 divides the 15 DTs into two categories. Compatible (C) patterns are DTs in which the F0 offset of the tonal target in S1 is close to the F0 onset of the tonal target in S2. Non-compatible (NC) patterns, however, have very different F0 values at the offset of the tonal target in S1 and the onset of the tonal target in S2 [32]. Fig 1 marks the C and NC tone combinations by dotted and solid lines, respectively. As shown NC DTs involve more rapid F0 changes, indicated by larger F0 ranges and steeper F0 slopes in the F0 contours, and require changes in the direction of F0 (from fall to rise or rise to fall) at the syllable boundary in more cases than in C DTs. Thus, the F0 contours for NC tone combinations are considered more complex than for C tone combinations.

**Table 1:** Compatible and non-compatible tone combinations in disyllabic words.

Compatible Tone Combinations (C)	Non-compatible Tone Combinations (NC)
T11 (¯ ¯), T14 (¯ \)	T12 (¯ /), T13 (¯ _)
T21 (/ ¯), T24 (/ \)	T22 (/ /), T23 (/ _)
T32 (_ /)	T31 (_ ¯), T34 (_ \)
T42 (\ /), T43 (\ _)	T41 (\ ¯), T44 (\ \)

Notes. T11 represents Tone 1 (T1) followed by T1. The symbols (¯, /, _, \) are schematic representations of the target F0 contours for the four tones (i.e., High, Rise, Low, Fall), respectively. Because T33 (_ _) is produced as T23 due to the Tone 3 sandhi rule, it is not listed in the table.

More complex F0 contours may be more difficult to produce than less complex F0 contours due to physiological constraints in pitch production. To achieve the tonal targets within the time frame of a syllable, production of tones with more complex F0 contours requires a higher velocity of F0 change and more changes in F0 direction. Yet there are physiological limits on the maximum speed of pitch change (i.e., acceleration and deceleration of F0) that can be produced. This maximum speed of pitch change is often approached by the adult speaker during the production of multisyllabic utterances [33]. Therefore, the production of more complex F0 contours imposes greater demands on the laryngeal system and is more susceptible to target undershoot [34].

Young children show significant differences from adults in the laryngeal morphology and physiology necessary for tone production. The larynx in infants and children is still undergoing anatomical changes and continues to undergo considerable development until 5 or 6 years of age [35, 36]. Infants’ vocal folds lack the distinctive layered structure found in adults’ vocal folds. The composition of their laryngeal musculatures does not reach the adult form until 16 years of age [37] and the length of the vocal folds continues to increase until around 20 years of age [36]. Children’s speech motor control is also different from that of adults. Young children produce slower [38, 39] and more varied [40, 41] speech movements. They are less mature in speech motor coordination, which involves the temporal and spatial control of the articulatory musculature [29, 39].

Given that young children’s larynges are not fully developed, their articulatory gestures are slower, and their control and coordination of the articulatory musculature are less mature, it was predicted that 2- to 6-year-old Mandarin children would show a gradual pattern of mastery of disyllabic lexical tone combinations and their tone production accuracy would be influenced by tonal context in the disyllabic words. Specifically, more complex F0 contour patterns (e.g., NC tone combinations) would be more difficult for children to produce. The goals of the present study were (1) to examine the developmental trends of children’s accuracy of the four tones produced in disyllabic words, (2) to investigate context effects on children’s coarticulated tone production accuracy and (3) to test the hypothesis that children’s tone production accuracy is related to F0 complexity.

## Method

The procedures followed the methods used in the studies developed by Wong and colleagues [3, 17, 18] and were approved by the Institutional Review Board of the Graduate Center of the City University of New York (IRB Number: 02-08-086).

### Collection of children’s and adults’ tone productions

#### Participants

Children. Forty-four Mandarin-speaking children (2 from working class and 42 from middle class families; 17M, 27F, age range: 2;1–6;7) growing up in monolingual Mandarin-speaking families in the U.S. participated in the study. Their mothers provided written informed consent for the participation of the children. Twelve children were 2-year-olds (C2, 2;0–2;11), 13 were three-year-olds (C3, 3;0–3;11), 11 were four-year-olds (C4, 4;0–4;11), and 8 were five years or older (C5+, 5;0–6;7). All children met the following criteria: (1) the child had unremarkable cognitive, social, physical, emotional, educational and speech and language history according to parental report; (2) family members and caregivers spoke only Mandarin to the child; (3) the child scored higher than the 20^th^ percentile rank in the total language score in the Chinese speech and language test—Language Disorder Scale of Preschoolers (LDSP, 學前兒童語言障礙評量表) [42] and lower than the 20^th^ percentile rank in the total language score in the English language test—Preschool Language Scale-4 (PLS-4) [43], (4) no language limitations or atypicalities were observed in the language sample collected; (5) the child passed hearing screening in both ears at 1k, 2 k and 4 kHz at 20 dB HL under headphones using conditioned play audiometry; and (6) the child did not have a history of chronic otitis media according to parental report. For the two youngest children (aged 2;1 and 2;2), language tests were attempted but no score was obtained. Both children did not respond to any of the test items in the English test. Their parents reported that the children had no exposure to English. One of the children had been in the U.S. for only two weeks. Neither child had the attention span to finish the LDSP, which was designed for children from 3;0–5;11. Parents of both children reported that the children had very good Chinese language skills. Language samples collected did not demonstrate any language issues. S1 Appendix provides background information for the children.

Though neither of the two language tests was designed for children with the cultural and linguistic backgrounds of those in this study, the tests were used to provide a general measure of the children’s language skills given that no appropriate test was available. The Chinese test was normed in Taiwan and the English test was standardized on monolingual English-speaking children. Twelve participants who took the test fell beyond the target age range of the LDSP (i.e., 3;0–5;11). Thus, the two-year-olds were compared to the norms of three-year-olds and the six-year-old children were compared to the norms of five-year-olds. The 44 children achieved a percentile rank of 21 to 93 for their total Chinese language scores. They all received much lower percentile ranks in their English total scores (range = 1–19 percentile), with a difference of 16 to 91 percentile ranks between the Chinese and English total scores.

Adults. Twelve mothers (age range = 27–45 years) of the child participants were recruited. They provided written consent for their participation. All mothers indicated Mandarin as their strongest and home language.

#### Stimuli

Fifteen familiar words representing the 15 disyllabic lexical tone combinations were chosen as the target stimuli based on familiarity pretesting with another group of 59 Mandarin-speaking children. Table 2 lists the stimuli.

**Table 2:** Stimuli.

Target Tone	Chinese Word	English Meaning	IPA	Pinyin
T11	西瓜/西瓜	watermelon	/ɕi1 kwa1/	xi1 gua1
T12	刷牙刷牙	brush teeth	/ʂwa1 ja2/	shua1 ya2
T13	喝水/喝水	drink	/xɤ1 ʂwei3/	he1 shui3
T14	鸡蛋/鷄蛋	egg	/ʨi1 tan1/	ji1 dan4
T21	毛巾/毛巾	towel	/mɑu2 ʨin1/	mao2 jin1
T22	蝴蝶/蝴蝶	butterfly	/xu2 tje2/	hu2 die2
T23	苹果/蘋果	apple	/pʰjəŋ2 kwo3/	ping2 guo3
T24	螃蟹/螃蟹	crab	/pʰɑŋ2 ɕje4/	pang2 xie4
T31	剪刀/剪刀	scissors	/ʨjɛn3 tɑu1/	jian3 dao1
T32	草莓/草莓	strawberry	/ʦʰɑu3 mei2/	cao3 mei2
T34	眼镜/眼鏡	glasses	/jɛn3 ʨjəŋ4/	yan3 jing4
T41	蛋糕/蛋糕	cake	/tan4 kɑu1/	dan4 gao1
T42	气球/氣球	balloon	/ʨʰi4 ʨʰjou2/	qi4 qiu2
T43	电脑/電腦	computer	/tjɛn4 nɑu3/	dian4 nao3
T44	电话/電話	telephone	/tjɛn4 xwa4/	dian4 hua4

### Procedures for collection of tone productions

The children attended one to two sessions of 30 minutes to one-and-a-half hours long, depending on the child’s attention span and the number of breaks needed. The child first completed a picture naming task in which they labeled the pictures two times. Simple questions such as “这是什么 [What is this]?”, “他在干吗 [what is s/he doing]?” were used to elicit productions. When the child failed to produce the target word, a toy object, a real object or gestures of the actions were presented. If the child still failed to produce the target word, semantic cues were given (e.g., “他很渴, 他在做什么 [He is very thirsty. What is he doing]?”). After the picture naming task, the Chinese language test was administered. Then a language sample using a picture book and free play was collected followed by a hearing screening. The English test was given at the end of the session. Mothers participating in the study filled out a language background questionnaire and labeled the pictures two times to the experimenter after the child had finished all the testing procedures. All the children’s and adults’ productions were recorded on a digital recorder in 16-bit PCM format at 44.1 kHz sampling frequency through a Shure dynamic microphone.

### Perceptual judgments of children’s and adults’ tones

To determine children’s accuracy in tone production, Mandarin-speaking adults were recruited to identify the children’s and adults’ tones.

#### Stimuli

Natural/unfiltered training stimuli. Forty-two unfiltered nonsense word stimuli were used for two training blocks to ensure that the judges could identify the tones without lexical knowledge. The first training block consisted of 12 (4 tones x 3 syllables) monosyllabic unfiltered nonsense words. The second block consisted of 30 (15 DTs x 2 words) disyllabic unfiltered nonsense words. All words were recorded onto a computer in a sound treated booth by a Mandarin-speaking female, and were normalized for intensity.

Filtered training stimuli. The 30 disyllabic nonsense words used in the third training block were low-pass filtered at 400 Hz to form the third training block to familiarize the judges with the task and the filtered stimuli in the experimental blocks. The filtering procedure retained the F0 information and eliminated most of the segmental information.

Filtered experimental stimuli. The stimuli for the experimental blocks were the target words produced by the children and mothers in the picture naming task. Non-target words, non-isolated productions, playful productions, tokens that were too loud (i.e., clipped) or too soft (e.g., unintelligible mumbles) and noisy tokens were excluded. If the first production of each lexical item could not be used, the second production was selected. Altogether, there were 552 usable child productions and 176 usable adult productions.

Adult and child productions were low pass-filtered at 400 Hz and 500 Hz, respectively, to eliminate lexical information [17, 44]. Child productions were filtered at a higher cut-off frequency because children tend to have a higher average F0. All filtered stimuli were normalized for intensity and grouped by speakers.

The judges listened to 12 blocks of mothers’ productions, 44 blocks of children’s productions and another 41 blocks of child and adult productions for another study. All stimuli were arranged into six experimental sets for six judgment sessions. The number of trials and the number of blocks for adult and child productions were comparable in each experimental set.

#### Judges

Three Mandarin-speaking adults between the age of 26 and 30 years were recruited as judges. They provided written informed consent for their participation. They all learned Mandarin from birth and reported Mandarin to be their dominant language.

#### Procedures

Each judge attended eight one-hour sessions. The stimuli were presented under headphones at a comfortable listening level by a customized computer program. In the training session, after the judge clicked “start” on the screen, the computer program randomly presented a sound file in the training block. In the experimental sessions, the blocks and the trials within blocks were presented in a random order. The judge could listen to a stimulus as many times as needed, then indicated his or her decision by clicking the number corresponding to the tones perceived (i.e., 1, 2, 3, 4 for monosyllabic lexical tones in the training blocks and 11, 12, 13 … for the disyllabic tone combinations). In the first session, the judges listened to the three training blocks in sequence. The three judges attained accuracy rates of 100%, 97%, and 100%, respectively, in the third training block. After the training blocks, the judges filled out a language background questionnaire and had a hearing screening. They all passed the hearing screening of 500, 1 k, 2 k, and 4 kHz at 20 dB HL in both ears. In the subsequent six sessions, the judges listened to the six experimental sets in a random order. In the last session, they rerated the experimental blocks they had listened to in the second session to establish intrajudge reliability.

## Results

### Data analysis

Because most group data violated the assumptions for parametric statistics, we performed non-parametric statistics in all analyses, except for using Pearson correlations (r) as a measure of the developmental trends in children’s tone accuracy. Accuracy of children’s and adults’ tones was determined by the judges’ correct identification of the target tones for the lexical items (intended tones) in filtered production. This method has been employed in a number of studies examining tone accuracy in children and adults (e.g., [3, 18]) and has been shown to reflect the acoustic characteristics of the correctly and incorrectly produced tones (e.g., if a correct or incorrect production was perceived as a rising tone, it had a rising pitch contour) [19, 45].

In the following sections, first, inter- and intra-judge reliability was examined. Then tone categorizations by the selected judges were analyzed from two perspectives. The first analysis approach followed the convention in the child literature on tone acquisition and determined tone accuracy on the basis of the four tones; that is, accuracy of the tones in each syllable was evaluated independently without taking into account the accuracy of the preceding or following tone in the disyllabic word. Judges’ identification accuracy of adults’ tones was investigated first to determine whether the judges were able to categorize the tones in filtered speech and to establish a reference to which children’s accuracy was compared. Then data on individual children’s overall accuracy, collapsing over the four tones, were presented to examine the developmental trend of tone accuracy. Next, children’s accuracy for the four tones was compared to adults’ by age group to determine whether children in any age group produced any of the four tones with adult-like accuracy (i.e., mastered the tone production). After that, context effects on children’s tone production accuracy were investigated.

In the second analysis approach, adult and child productions were analyzed on the basis of 15 DTs. That is, correct productions were defined as correct identification of both tones in the disyllabic words by the judges. First, adults’ accuracy rates of DTs were analyzed and used as a reference for children’s productions. Then children’s overall accuracy rates across the 15 DTs and on each of the DTs were presented as a function of age. After that, children’s accuracy rates of the 15 DTs were compared to adults’ to determine whether children had mastered any of the DTs. Finally, accuracy rates of C vs. NC DTs were compared to test the hypothesis that more complex F0 contours (i.e., F0 contours in NC tones) are more difficult for children to produce.

### Interjudge reliability

Interjudge agreement was examined using overall accuracy rates (summing across all DTs in 30 words) of the 56 participants (12 adults and 44 children) and overall accuracy rates of the DTs by the 44 child speakers as the sampling variables. Kendall’s Coefficient of Concordance was used to establish the overall agreement among the three judges. Spearman’s rank-order correlations were computed to examine the agreement between each pair of judges.

The three judges were highly correlated on their rankings of the overall accuracy scores for the 56 speakers, [W(N = 3, df = 55) = .907, χ^2^ = 149.58, p < .001] and for the 44 children, [W(N = 3, df = 43) = .838, χ^2^ = 108.15, p< .001]. Spearman correlations of each pair of judges were also quite high on overall accuracy rates for all 56 speakers (.893 to .936) and for the 44 children (.831 to .889).

### Intrajudge reliability

Spearman’s Rank-Order correlation coefficients were computed to examine the test-retest reliability of each of the three judges on the overall scores of the 30 disyllabic words of all the speakers and the overall scores of the 30 words of the child speakers. All judges demonstrated very strong test-retest reliability with Spearman correlations ranging from 0.904 to 0.940 for the tones of all speakers and from 0.856 to 0.904 for children’s tones.

### Accuracy in the production of the four Mandarin tones: Age and context effects

#### Judged accuracy of adults’ productions

Adults’ tone productions were judged with high accuracy in both syllables (S1 and S2) and in both contextual conditions (C and NC), indicating that the judges were able to identify the target tones in filtered disyllabic stimuli without the aid of lexical information. The mean overall accuracy rates collapsing across the four tones were 99% (SD = 2%) in S1, 98% (SD = 2%) in S2, 96% (SD = 6%) in C contexts (i.e., being preceded or followed by a C tone), 98% (SD = 3%) in NC contexts (i.e., being preceded or followed by a NC tone). The mean accuracy for each of the four tones with all contexts collapsed were 97% (SD = 5%), 100% (SD = 0%), 98% (SD = 3%), and 99% (SD = 3%) for T1, T2, T3 and T4, respectively. The accuracy rates of the four tones in each of the context types tested (i.e., S1C, S1NC, S2C, S2NC) were also very high, ranged from 94%-100%.

#### Judged accuracy of children’s productions

**Fig 2:**
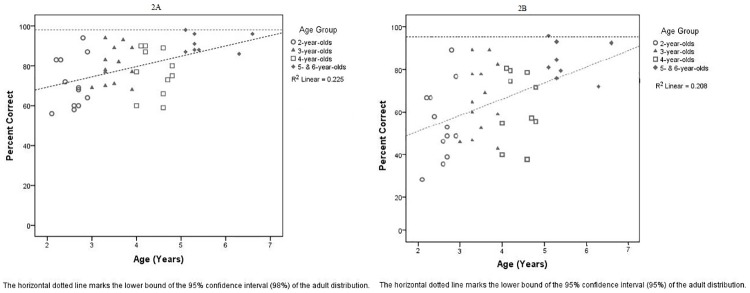
Development of Mandarin tones in children. (A) Development of the Overall Accuracy Rates of the Four Tones by Children. (B) Development of the Overall Accuracy Rates of the Disyllabic Tone Combinations by Children.

Children’s tone productions were judged less accurately than adult productions. Fig 2A shows a scatterplot of the 44 children’s overall accuracy in production, collapsing over the four tones in both syllable positions and in both C and NC combinations. The horizontal dotted line represents the lower bound of the 95% confidence interval of the adults’ scores (i.e., 98% accuracy). Overall, children’s accuracy rates improved with age as indicated by a significant positive relation between age and the overall accuracy, [r_s_ (N = 44) = .470, p = .001]. Age accounted for 23% of the variance in children’s accuracy of the four tones. The data for individual children showed that more children in the oldest age group approached the adult overall accuracy rate. Inter-subject variability within age groups decreased substantially for children over five years of age.

**Fig 3:**
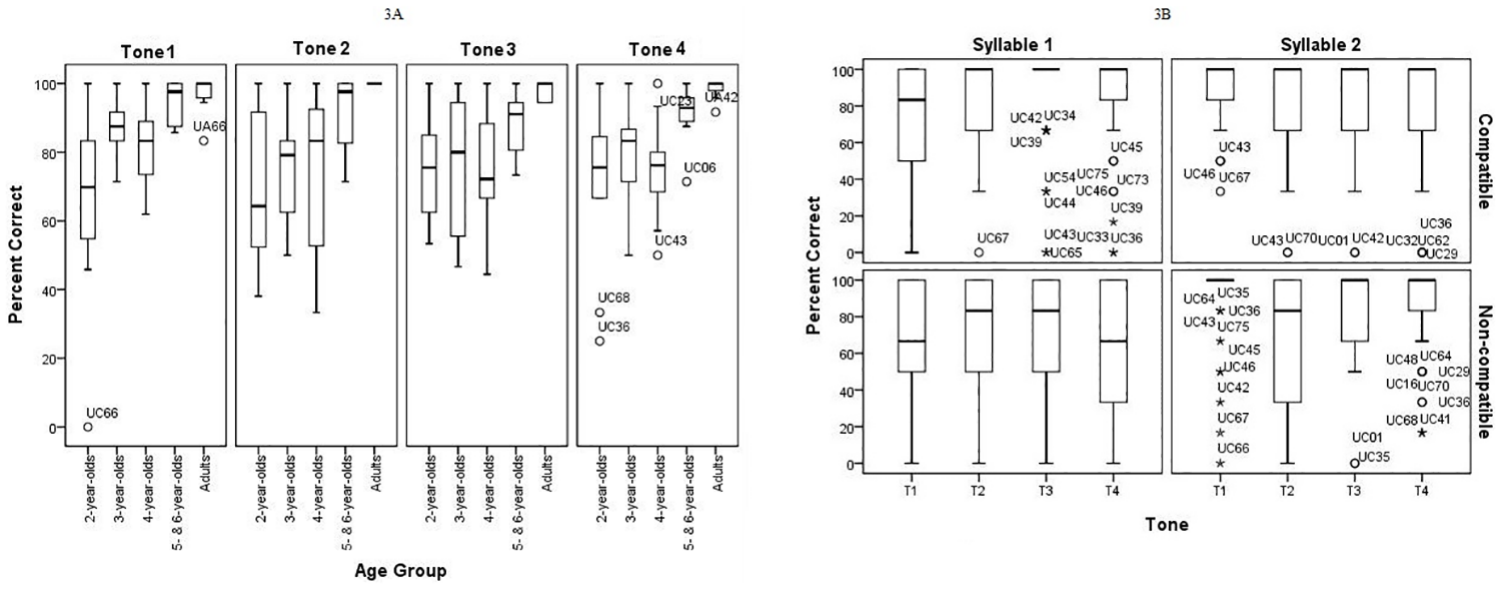
Percent correct of the four tones by age group. Note. “o” represents outliers while “*” represents extreme values.

When comparing child and adult tone accuracy, except for T1 by 5- and 6-year-olds, children in all age groups produced the four tones with significantly lower accuracy than adults, with mean differences from adult accuracy ranging from 8% to 30%. Differences between children and adults were statistically highly reliable (p < .008) (Table 3). The mean difference in T1 accuracy between adults and 5- and 6-year-old children was 3% and did not reach statistical significance, suggesting adult-like accuracy. Fig 3A shows the overall accuracy rates of the individual tones collapsing across contexts by age group. Overall, children’s productions of the four tones demonstrated larger variability than adults. Accuracy improved and variability within age group decreased in older children, particularly in 5- and 6-year-olds.

**Table 3:** Accuracy (mean percent judged as intended) of the four tones collapsing across all contexts.

Tone	$\bar{\text{X}}$ (%)	SD (%)	U	z	p	Effect size (r)
All Children						
T1	81	19	93.5	-3	0.001**	0.46
T2	76	20	54	-4	0.000**	0.58
T3	79	17	58	-4	0.000**	0.56
T4	78	17	46	-4	0.000**	0.59
2-year-olds						
T1	67	27	18	-3	0.001**	0.65
T2	70	22	12	-4	0.000**	0.79
T3	76	16	16	-3	0.001**	0.69
T4	71	22	9	-4	0.000**	0.78
3-year-olds						
T1	87	8	24	-3	0.003**	0.60
T2	75	16	12	-4	0.000**	0.79
T3	77	20	14	-4	0.000**	0.72
T4	80	16	16	-4	0.000**	0.71
4-year-olds						
T1	81	11	13	-3	0.001**	0.70
T2	75	24	6	-4	0.000**	0.85
T3	76	17	16	-3	0.001**	0.67
T4	75	15	9	-4	0.000**	0.77
5- and 6-year-olds						
T1	94	7	39	-1	0.446	0.17
T2	91	11	24	-3	0.008**	0.59
T3	88	9	12	-3	0.003**	0.66
T4	91	9	13	-3	0.003**	0.66

Statistics report nonparametric results of comparisons of each group with adult performance using the Mann-Whitney U Test of Independent Group Differences.

Notes:

** represents significance level < .01.

Small effect sizes: 0.10–0.29, medium effect sizes: 0.30–0.49, large effect sizes: 0.50–0.69, much larger than typical effect sizes: 0.70 or above.

Unlike adults, judged accuracy of children’s tone productions was influenced by syllable position and tonal contexts. Counter to our prediction, Wilcoxon signed-ranks tests showed that children as a group produced tones less accurately in S1 than in S2, [z (N = 44) = -2.012, p = .04], with mean accuracy = 77% (SD = 14%) and 81% (SD = 13%), respectively (Table 4 upper left hand cluster), mainly due to the large difference in performance on Tone 1.

**Table 4:** Context effects on children’s tone production accuracy.

	Mean (%)	z	p	Effect size (r)		Mean (%)	z	p	Effect size (r)
**S1 vs. S2**					**C vs. NC**				
All tones	77 vs. 81	-2.012	0.044*	0.21	All tones	71 vs. 60	-3.332	0.001**	0.36
Tone 1	75 vs. 88	-3.523	0.000**	0.38	Tone 1	83 vs. 81	-0.272	0.786	0.04
Tone 2	80 vs. 73	-1.459	0.145	0.16	Tone 2	83 vs. 70	-2.91	0.004**	0.44
Tone 3	77 vs. 81	-0.875	0.382	0.10	Tone 3	84 vs. 76	-1.846	0.065	0.28
Tone 4	74 vs. 82	-1.494	0.135	0.16	Tone 4	82 vs. 75	-1.713	0.087	0.26
**S1C vs. S2C**					**S1C vs. S1NC**				
All tones	84 vs. 82	-0.821	0.412	0.09	All tones	84 vs. 71	-4.450	0.000**	0.47
Tone 1	76 vs. 89	-2.184	0.029*	0.25	Tone 1	76 vs. 74	-0.731	0.465	0.08
Tone 2	86 vs. 79	-1.455	0.146	0.17	Tone 2	86 vs. 75	-2.236	0.025*	0.26
Tone 3	87 vs. 80	-0.475	0.634	0.06	Tone 3	87 vs. 71	-2.878	0.004**	0.33
Tone 4	83 vs. 78	-0.473	0.636	0.05	Tone 4	83 vs. 64	-2.647	0.008**	0.29
**S1NC vs. S2NC**					**S2C vs. S2NC**				
All tones	71 vs. 81	-3.311	0.001**	0.35	All tones	82 vs. 81	-0.614	0.539	0.07
Tone 1	74 vs. 89	-2.813	0.005**	0.32	Tone 1	89 vs. 89	-1.057	0.290	0.12
Tone 2	75 vs. 68	-0.833	0.405	0.09	Tone 2	79 vs. 68	-1.892	0.058	0.21
Tone 3	71 vs. 81	-2.052	0.040*	0.24	Tone 3	80 vs. 81	-0.07	0.944	0.01
Tone 4	64 vs. 85	-2.734	0.006**	0.30	Tone 4	78 vs. 85	-1.898	0.058	0.22

Notes: “S1” represents syllable 1, the first syllable. “S2” represents syllable 2, the second syllable. “C” stands for compatible combinations. “NC” stands for non-compatible combinations.

* indicates significance at .05 level.

** indicates significance at .01 level.

Effect size of 0.10–0.29 represents small effect size. 0.30–0.49 represents medium effect sizes, 0.50–0.69 represents large effect sizes and 0.70 and above represents much larger than typical effect sizes.

As predicted, children as a group made significantly more errors on tones in NC than C contexts [z (N = 44) = -3.332, p = 0.001], mean accuracy = 60% (SD = 21%) vs 71% (SD = 21%), respectively (Table 4 upper right hand cluster). Further analysis of children’s overall performance in the four contextual conditions (i.e., S1C, S2C, S1NC, S2NC) with Friedman’s ANOVA showed an overall effect of syllable position and tone compatibility [χ^2^ (3) = 26.65, N = 43, p < .001]. Post hoc pairwise analyses using Wilcoxon Signed-Ranks tests showed that the overall production accuracy of tones in the first syllable of NC disyllable combinations (S1NC) was significantly lower than in the other three contexts, S1NC < S1C [z (N = 44) = -4.450, p < .001, r = -.474], S1NC < S2C [z (N = 43) = -2.995, p = .003, r = -.319], S1NC < S2NC [z (N- = 44) = -3.311, p = .001, r = -.353]. No other comparisons reached significance.

Children’s accuracy for the individual tones was affected by different tonal contexts to different degrees. Fig 3B shows box and whisker plots of accuracy of children’s four tones in different syllable positions and compatibility contexts, collapsed over all four age groups. T1 accuracy was affected significantly by syllable position [z (N = 42) = -3.523, p < .001, Wilcoxon signed ranks tests] and was produced less accurately in S1 than in S2 in both C and NC combinations (Table 4, Fig 3B). T2 was produced with significantly lower accuracy in NC contexts than in C contexts, [z (N = 43) = -2.91, p = .004] overall, and especially in S1 context. For T3 and T4, children made significantly more errors when the tones were in S1 followed by a NC tone in S2 (lower left quadrant of Fig 3B).

### Accuracy in terms of the 15 disyllabic lexical tone patterns (DTs)

In this part of the analyses, the DTs were treated as one unit. Thus, correct production was defined as correct identification by the judges of the target tones in both syllables.

#### Judged accuracy of adults’ production of DTs

The judges identified the adults’ DTs with high accuracy (range = 82% to 100%). Three T11 adult productions were incorrectly judged by one of the three judges and one adult T11 production was judged as T13 by all three judges, resulting in the lowest accuracy rate of 82% for T11. Three judgment errors were made for adults’ T13, yielding the second lowest rate of 92%. Two judgment errors were made for T14, T41, and T43 resulting in an accuracy rate of 94%. All other adult DTs were identified with 100% accuracy.

#### Judged accuracy of children’s production of DTs

**Fig 4:**
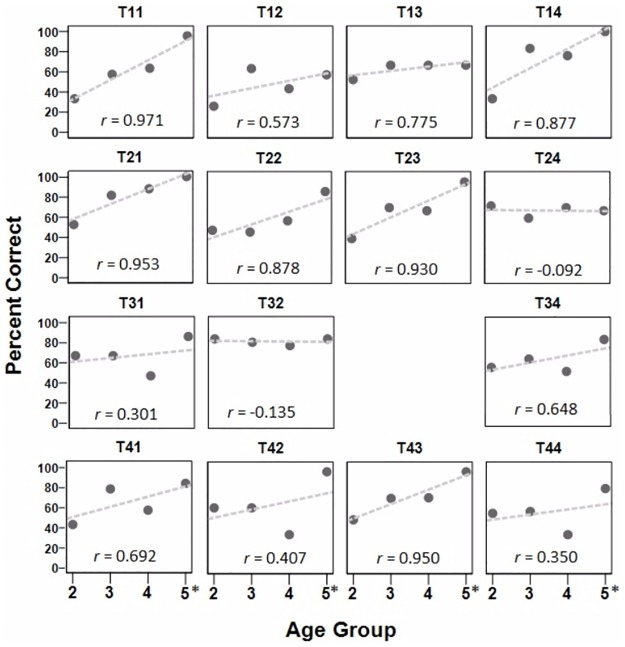
Development of the accuracy rates of the 15 disyllabic tone combinations by children. Note. 5* represents five- and six-year-old children.

Children’s overall accuracy, averaging across the 15 DTs, was subjected to correlational analysis. Fig 2B presents the scatter plot of children’s overall accuracy rates as a function of age. There was a significant positive relation between age and DT accuracy, [r_s_ (N = 44) = .453, p = .002] with age accounting for approximately 21% of the variability in the children’s performance. Similar to Fig 2A, the scatterplot shows that children between 2 and 4 years of age varied greatly in their ability to produce DTs accurately. Due to the lack of variations in the accuracy of each tone combination by each child (accuracy = 0%, 33%, 67%, or 100%), the three judges’ identification accuracy of the 15 DTs produced by the children in each age group was computed and presented in scatterplots to examine the developmental trends of tone accuracy in each tone combination. Fig 4 presents children’s performance on individual DTs by age groups, with a regression line indicating the rate of development over age. Positive correlations between accuracy rates and age were found for all 15 DTs, except T24 and T32. The large variations (range from -0.092 to .971) in the correlation coefficients (*r*) suggested that different DTs developed at different rates.

Mann-Whitney U tests of independent groups were conducted to determine if children’s production accuracy on the DTs differed from that of adults. Averaging over all 15 DTs, children produced disyllables significantly less accurately than adults [U (N = 56) = 4.5, p = < .001, r = -.693]. Comparing tone accuracy in each child group with adults showed that even 5- and 6-year-old children’s overall accuracy of DTs was significantly lower than that for adults [U (N = 20) = 4.500, p = .001, r = -.757].

In terms of the individual DTs, Table 5 lists the lower bound of adults 95% confidence interval for each DT combination and the percentage of children in each age group whose accuracy rates on the DTs were comparable to adults (i.e., equal to or higher than the lower bound of the 95% confidence interval of the adults’ score distributions). In general, the number of children who produced the DTs with adult-like accuracy increased with age. If “mastery” of a DT combination is defined as a majority (51%) of children showing adult-like accuracy (shown in bold), the children as a group had mastered six of the seven C tones and only one of the eight NC tones (Table 5). T21 and T32 were mastered by the most children (71%). Only two C tones and one NC tones were produced with adult-like accuracy by the majority of two-year-olds, suggesting that they had difficulty with both C and NC tone combinations. T24 and T32 were the easiest as most (over 70%) two-year-olds produced them with adult accuracy rates, while T12 and T14 were the most difficult as none of the two-year-olds produced them with adult-like accuracy. Three- and four-year-old children mastered more C tone combinations, but not more NC combinations, than 2-year-olds, suggesting that while the production of C disyllables was improving, NC combinations remained difficult for three and four-year-old children. Among the C tones, T14 was the easiest and was produced with adult-like accuracy by the most (83%) three-year-old children. Non compatible T22 was the most difficult for the three-year-olds; only 27% of the children produced it with adult-like accuracy. Four-year-old children mastered four C tones and one NC tone. T21 was produced with adult-like accuracy by the most (82%) four-year-olds, while T42, a compatible tone combination linking two dynamic tones, was produced with adult-like accuracy by the fewest (10%) four-year-olds. For both 3- and 4-year-olds, most of the NC tones were among the tones that were produced by the fewest number of children with adult-like accuracy (Table 5). Five- and six-year-old children had mastered 13 tone combinations: 6 C and 7 NC. T11, T14, T21 were produced with adult-like accuracy by all 5- and 6-year-old children. Most tones that were produced by the fewest number of 5- and 6-year-olds with adult-like accuracy were NC tones, except for T24, which is a C combination of two dynamic tones.

**Table 5:** Percentage of children in each age group whose accuracy rates for the 15 disyllabic tone combinations were adult-like.

		T11	T12	T13	T14	T21	T22	T23	T24	T31	T32	T34	T41	T42	T43	T44
		C	NC	NC	C	C	NC	NC	C	NC	C	NC	NC	C	C	NC
A	Adult 95%CI ^a^	60.9	95.6^b^	82.1	86.2	95.6^b^	95.6^b^	95.6^b^	95.6^b^	95.6^b^	95.6^b^	95.6^b^	82.2	95.6^b^	86.2	95.6^b^
C2	% within 95%CI ^c^	22	0	43	0	29	33	33	**71**	**55**	**75**	25	36	40	22	36
C3	% within 95%CI	**55**	30	30	**83**	67	27	**64**	44	36	**60**	42	**67**	40	**58**	46
C4	% within 95%CI	**64**	20	50	43	**82**	40	**60**	**55**	30	**70**	36	40	10	50	20
C5	% within 95%CI	**100**	43	**57**	**100**	**100**	**71**	**86**	38	**57**	**83**	**75**	**63**	**88**	**88**	**63**
All ^d^	% within 95%CI	**59**	22	44	**52**	**71**	40	**62**	**51**	44	**71**	42	50	42	**54**	40

^a^ lower bound of the 95% confidence interval of adults’ accuracy rate

^b^All adults’ productions were judged with 100% accuracy. Thus the lower bound of the 95% confidence interval of the adults’ scores was set at 95.6%.

^c^ Percent of children whose accuracy rates of disyllabic tone sequences were higher than or equal to the lower bound of the 95% confidence interval of the adults’ scores.

^d^ All children as a group (i.e., children from two to six years old)

The numbers in bold indicate that more than 50% of the children in the group attained adult-like tone accuracy.

In sum, these patterns suggest that very young children had difficulty with both C and NC tones. As children grew older, they mastered more C tones, but NC remained difficult. Even 5- and 6-year-olds had more difficulty with NC tones than C tones. Further analysis with Wilcoxon Signed Ranks tests demonstrated that children produced NC DTs significantly less accurately than C DTs, [N = 44, z = -3.169, p = .002, r = -.338].

Analysis of the major substitution patterns (judges’ use of another DT combination to categorize the intended DT combination) of children’s productions showed that children tended to modify the tones in S1, particularly in NC combinations. Seventeen major substitution patterns (substitution patterns that accounted for more than 10% of the total judgments for the target DT combination) were identified for 2- to 4-year-old children. Eleven of the 17 substitutions involved a misidentification of the S1 tone by the judges in three C tone combinations (T11, T14, T43) and 5 NC tone combinations (T23, T31, T34, T41, T44). Four of the six major substitution patterns for the NC combinations involved the tone in S1 being identified as a tone with an F0 offset value closer to the S2 F0 onset value (T31→T21, T34→T24, T41→T11, T44→T14), changing the NC combinations to C combinations and showing anticipatory coarticulation effect. S2 Appendix shows the confusion matrices for the adults’ and children’s productions of disyllabic tone combinations.

## Discussion

Counter to findings in early studies claiming that lexical tones are acquired well before segmental production is mastered, results of the present investigation confirm the findings in recent studies on monosyllabic Mandarin tone productions that acquisition of Mandarin lexical tone is a lengthy and gradual process that spans more than five years. Most preschool children have not mastered Mandarin tone production in disyllabic words before six years of age. As children get older, their tone accuracy in disyllabic words improves, more tones in more contexts are produced with adult-like accuracy, and more children in the same age group attain tone accuracies closer to those of adults. Accurate production of a lexical tone sequence requires phonetic learning, motor learning, and implementation of the motor plan with precise, efficient and temporally coordinated laryngeal and supralaryngeal articulatory gestures. Given that these systems are still developing in young children, it is not surprising that adult-like tone production takes time to master.

Substantial cross-subject variability was observed in children’s disyllabic lexical tone productions. Overall, inter-subject variability declined in older age groups, with the most substantial decrease in five- to six-year-old children. Developmental variability in speech sound production has been widely reported in the literature and was proposed as an indication of neuromotor immaturity [41, 46]. As children get older, they progressively refine their speech motor control [47]. Thus, inter-subject and intra-subject variability decreases with age.

Another theoretical perspective on children’s variability in speech production claims that speech variability is a result of an adaptive mechanism in the developing child [48]. According to this view, young children need to remain flexible in their articulatory movements so as to constantly explore and learn different articulatory patterns to achieve the same articulatory goal in response to the anatomical and biomechanical changes in their speech production system [38, 41]. This can explain why children in younger age groups exhibited more inter-subject variability in tone production accuracy.

The findings of the current study show significant context effects on children’s tone production. Similar results have also been reported in Mandarin tone production in adult heritage Mandarin speakers [21] and Mandarin tone perception acquisition with adult second language learners of Mandarin [20, 49]. Overall, T1 is more difficult for children to produce when it occurs in the first syllable, suggesting that reaching a high and level target at the onset of production seems to be more challenging. T2 is more difficult to produce when it is preceded or followed by a NC tone. Given that T2, the rising tone, is a dynamic tone that is produced with a relatively large F0 range and that a rising F0 contour is more difficult for young children to produce than a falling F0 contour [3, 19, 50], producing T2 in a NC tonal context could increase articulatory difficulties for children. For T3 and T4, children make more errors when these two tones precede a NC tone. Possible reasons for children making errors in S1 for NC tone combinations are presented below. Given the context effects on tone accuracy, order of acquisition of the four tones is dependent on context and tone accuracy in one context may not be predictive of the accuracy of the same tone in another context. It appears that overall T1 is acquired earlier than the other three tones in disyllabic contexts, as it is the only tone produced with adult-like accuracy by five and six year old children (Fig 3 and Table 2).

The findings support the hypothesis that more complex F0 contours are more difficult for children to produce: children in the study produced the four tones in NC contexts with significantly lower accuracy than in C contexts (Table 4), NC disyllabic F0 contours were produced with significantly lower accuracy than C disyllabic F0 contours (Table 4). In addition, the majority of the tone combinations that were produced by the fewest number of children with adult-like accuracy were NC tone combinations (Table 5). Given that more complex F0 contours involve more rapid changes in velocity and direction of F0, the data support the claim in previous tone studies that tone acquisition might be related to the maturation of laryngeal control and limited by physiological constraints.

The finding that children make more errors in the first than the second syllable does not refute our hypothesis that tone combinations with more complex F0 contours are more difficult. First, overall accuracy of the tones in S1 was only marginally significantly lower than the overall accuracy of the tones in S2, with a small effect size (Table 4). More importantly, further analyses revealed that tones in S1C, S2C and S2NC contexts were produced with comparable accuracy and only tones in S1NC contexts were produced with significantly lower accuracy rates. The major substitution patterns in children’s errors in producing DTs shed some light on why children made more errors in S1 when producing NC tones and why tones in S1C, S2C and S2NC contexts were produced with comparable accuracy. The error patterns suggested that when children made errors in producing NC tones, they tended to modify the F0 contour in the first syllable such that the F0 offset in the first syllable became closer to the F0 onset of the tone in the second syllable (e.g., T31 (LH) → T21 (RH), T34 (LF) → T24 (RF); T41 (FH) → T11 (HH), T44 (FF) → T14 (HF)). If the F0 contour of the first tone in the NC tone combination was modified and became compatible to the second tone, the tone in the second syllable would no longer be in a NC context but would have become compatible with the F0 contour in S1. Therefore, it is not surprising that the accuracy in S2NC was comparable to that of S2C and S1C.

The finding that children made more errors in the tones in S1 when it was followed by a NC tone also suggests that children’s difficulties with more complex F0 contours are associated more with production difficulties than perceptual difficulties. In adults’ disyllabic lexical tone productions, due to little anticipatory coarticulation in the first syllable, the F0 contours of the same tone in the first syllable are similar to each other when preceding the four tones in both C and NC conditions. Thus, children receive similar acoustic input of the tones in the first syllable of the disyllabic word, regardless of the tone in the second syllable. Therefore, children’s lower accuracy on the first tones in NC versus in C conditions is not likely due to perceptual difficulty. Also, in adult productions, the F0 contours of the same tones varied substantially (further from canonical forms) and were more complex in the second syllable, particularly when it was NC with the tone in the first syllable. Comparable accuracy found in children’s productions in the first and second syllable in compatible contexts and in the second syllable in non-compatible contexts does not support a perceptual account for the difficulties.

The finding that children made more tone errors in S1 in NC contexts and tended to shift the F0 offset of the F0 contour in S1 to meet the F0 onset of the tone in S2 in NC contexts suggests that children produced more anticipatory coarticulation than adults. The hypotheses proposed by previous studies on children’s segmental coarticulation concerning the use of syllable vs. phoneme as a production unit in children are not applicable to tone coarticulation in children. In adults, the production unit for tone is the syllable [7] and tones function as phonemes in tone languages. Analysis of adult and children’s syllable duration showed that children produced syllable durations in different contexts (i.e., S1, S2, S1C, S1NC, S2C, S2NC) comparable to adults, with syllable duration significantly shorter in S1 (mean = 0.27s and 0.28s for adults and children, respectively) than in S2 (mean = 0.37s and 0.39s, for adults and children, respectively). No differences were found in the syllable duration of S1C vs. S1NC (mean = 0.26s vs. 0.27s in adult productions, and 0.27s vs. 0.28s in children’s productions), or S2C vs. S2NC (mean = 0.36s vs. 0.37s in adult productions, and 0.40s vs. 0.39s in children’s productions).

The finding that children produced the tone in S1 correctly when it was followed by a C tone, but modified the pitch contour of the same tone to reduce the F0 difference between the tone offset in S1 and the tone onset in S2 when it is followed by a NC tone suggests that the simplification of the F0 contours in NC tones is to reduce the articulatory demands for producing complex F0 contours in NC tones in the disyllabic time-frame. Thus, anticipatory coarticulation of tones in children’s productions is likely a by-product of children’s inability to produce complex F0 contours. Given that more anticipatory coarticulation was observed in producing NC tones than C tones, the data seem to support the view that the amount of anticipatory coarticulation in children is dependent on gestural complexity (Repp, 1986). Tones with more complex F0 contours are subject to more anticipatory coarticulation. If excessive coarticulation in children is related to articulatory demands in the production, it is not surprising that different degrees of coarticulation were reported in previous studies that examined children’s segmental coarticulation using different segmental combinations.

## Conclusions

In summary, acquisition of Mandarin lexical tones is a protracted process that takes more than six years. Despite significant improvement in children’s tone accuracy over time, children as old as six years of age have not yet mastered all disyllabic lexical tone combinations in an adult-like manner. Large intra-group variability is observed in 2- to 4-year-olds but decreases substantially in 5- to 6-year-olds. Different Mandarin tones and tone combinations develop at different rates and are dependent on syllable and tone context. T1 is mostly affected by syllable position. T2 is affected by the compatibility of the tonal context. T3 and T4 are at their most challenging when they occur in the first syllable of non-compatible disyllabic tones. In general, more complex fundamental frequency contours are more difficult for children to produce. When producing more complex contours such as in non-compatible tone combinations, children exhibit more anticipatory coarticulation; they tend to modify the offset of the first syllable in a disyllabic tone to reduce the F0 gap between the F0 offset of the first tone and the F0 onset of the second tone. These results call into question the prevailing assumption that suprasegmental features are acquired before segmental features in children and suggest that the rate and order of acquisition of tone is dependent on articulatory complexity and the maturity of the speech motor coordination in children. Given the importance of lexical tone in tone languages and the lengthy time course of development, assessment and treatment of lexical tones should not be neglected in clinical protocols. It is also important to take context into account when investigating, evaluating, and treating tone development and phonological disorders in both research and clinical settings.

## Supporting information

S1 Appendix Background information of child participants. Note. ^a^Percentile rank score in the Chinese speech and language test—Language Disorder Scale of Preschoolers (LDSP, 學前兒童語言障礙評量表). ^b^Percentile rank score in the English language test—Preschool Language Scale-4 (PLS-4). ^c^Number of months the child lived in China or Taiwan. ^d^English schools were in the Chinatowns of New York with a large Chinese population. ^e^Information in parentheses indicates the number of months attending English schools.
(DOCX)

S2 Appendix Judges’ responses to adults’ productions of disyllabic tone combinations. A. Judges' Responses to adults’ disyllabic tones Productions. B. Judges' responses to 2- to 4-year-old children's productions of disyllabic tone combinations. Note: The cells in black mark correct identification of the tones. The light shaded cells mark error patterns that constitute more than 10% of the total number of trials for the tone.
(DOCX)
