# Supplementary material for: Phonetic complexity affects children’s Mandarin tone production accuracy in disyllabic words: A perceptual study
Source: PLoS One. 2017 Aug 14;12(8):e0182337. doi: 10.1371/journal.pone.0182337 (PMC5555563; doi:10.1371/journal.pone.0182337)
Supplement: S1 Appendix — Note. aPercentile rank score in the Chinese speech and language test—Language Disorder Scale of Preschoolers (LDSP, 學前兒童語言障礙評量表). bPercentile rank score in the English language test—Preschool Language Scale-4 (PLS-4). cNumber of months the child lived in China or Taiwan. dEnglish schools were in the Chinatowns of New York with a large Chinese population. eInformation in parentheses indicates the number of months attending English schools. (DOCX) [file pone.0182337.s001.docx]

Appendix A. Background information of child participants

|  | **ID #** | **Age** | **Gender** | **Chinese Percentile Scores^a^** | **English Percentile Score^b^** | **Months in China /Taiwan^c^** | **Type of School** |
| --- | --- | --- | --- | --- | --- | --- | --- |
| 2-year-olds | UC67 | 2;1 | M | NA | NA | 0 | Chinese Day Care |
|  | UC66 | 2;2 | F | NA | NA | 25 | Not attending School |
|  | UC38 | 2;4 | F | 21 | 1 | 0 | Chinese Day Care |
|  | UC34 | 2;5 | F | 85 | 5 | 1 | Chinese Day Care |
|  | UC36 | 2;7 | M | 51 | 1 | 24 | Not attending school |
|  | UC68 | 2;7 | M | 47 | 3 | [3](#RANGE!_ftn4) | Not attending school |
|  | UC01 | 2;8 | F | 79 | 4 | 0 | Chinese Day Care |
|  | UC35 | 2;8 | F | 51 | 6 | 0 | Chinese Day Care |
|  | UC46 | 2;8 | M | 54 | 1 | 8 | Chinese Day Care |
|  | UC32 | 2;9 | F | 37 | 1 | 12 | Chinese Preschool |
|  | UC16 | 2;11 | M | 70 | 1 | 0 | Chinese Day Care |
|  | UC44 | 2;11 | F | 64 | 1 | 0 | Not attending school |
| 3-year-olds | UC42 | 3;0 | F | 59 | 1 | 18 | Chinese Day Care |
|  | UC07 | 3;3 | F | 70 | 8 | 33 | Chinese Preschool |
|  | UC10 | 3;3 | F | 82 | 1 | 0 | Chinese Preschool |
|  | UC47 | 3;3 | F | 92 | 1 | 3 | English preschool^d^ (1 month)^e^ |
|  | UC48 | 3;3 | M | 39 | 6 | 0 | Chinese Preschool |
|  | UC65 | 3;4 | F | 51 | 6 | 24 | English School (3 months) |
|  | UC33 | 3;6 | M | 30 | 2 | 0 | Chinese Preschool |
|  | UC62 | 3;6 | M | 93 | 16 | 0 | Not attending school |
|  | UC26 | 3;7 | M | 70 | 1 | 43 | Chinese Preschool |
|  | UC17 | 3;8 | M | 78 | 3 | 6 | English school (12 months) |
|  | UC29 | 3;11 | F | 45 | 1 | 0 | Not attending School |
|  | UC39 | 3;11 | F | 78 | 1 | 43 | English preschool (2 months) |
|  | UC55 | 3;11 | M | 89 | 14 | 0 | Chinese Day Care |
| 4-year-olds | UC41 | 4;0 | F | 67 | 9 | 2 | Chinese Preschool |
|  | UC45 | 4;0 | F | 86 | 1 | 42 | Not attending school |
|  | UC23 | 4;1 | M | 35 | 1 | 0 | Chinese Preschool |
|  | UC28 | 4;2 | F | 26 | 3 | 27 | Chinese Preschool |
|  | UC72 | 4;2 | M | 69 | 1 | 30 | English school (14 months) |
|  | UC43 | 4;7 | M | 35 | 1 | 31 | Not attending school |
|  | UC70 | 4;7 | M | 85 | 1 | 29 | English school (2 weeks) |
|  | UC73 | 4;7 | M | 55 | 1 | 0 | Chinese Day Care |
|  | UC75 | 4;8 | F | 30 | 5 | 5 | English school (12 months) |
|  | UC56 | 4;9 | F | 35 | 16 | 6 | English School (7 months) |
|  | UC64 | 4;10 | F | 30 | 8 | 0 | English School (8 months) |
| 5- and 6-year-olds | UC22 | 5;1 | F | 67 | 3 | 19 | Chinese Preschool |
|  | UC54 | 5;1 | F | 76 | 1 | 3 | Chinese Preschool |
|  | UC06 | 5;3 | F | 62 | 1 | 0 | Chinese Preschool |
|  | UC08 | 5;4 | F | 51 | 1 | 57 | Not attending school |
|  | UC53 | 5;4 | F | 48 | 2 | 2 | Chinese Preschool |
|  | UC18 | 5;5 | F | 29 | 13 | 0 | English School (17 months) |
|  | UC57 | 6;3 | F | 62 | 19 | 24 | English School (10 months) |
|  | UC52 | 6;7 | M | 92 | 1 | 78 | Not attending (In US: 1 month) |

Note. ^a^Percentile rank score in the Chinese speech and language test—Language Disorder Scale of Preschoolers (LDSP, 學前兒童語言障礙評量表). ^b^Percentile rank score in the English language test—Preschool Language Scale-4 (PLS-4). ^c^Number of months the child lived in China or Taiwan. ^d^English schools were in the Chinatowns of New York with a large Chinese population. ^e^Information in parentheses indicates the number of months attending English schools.
