# Supplementary material for: Phonetic complexity affects children’s Mandarin tone production accuracy in disyllabic words: A perceptual study
Source: PLoS One. 2017 Aug 14;12(8):e0182337. doi: 10.1371/journal.pone.0182337 (PMC5555563; doi:10.1371/journal.pone.0182337)
Supplement: S2 Appendix — A. Judges' Responses to adults’ disyllabic tones Productions. B. Judges' responses to 2- to 4-year-old children's productions of disyllabic tone combinations. Note: The cells in black mark correct identification of the tones. The light shaded cells mark error patterns that constitute more than 10% of the total number of trials for the tone. (DOCX) [file pone.0182337.s002.docx]

*Appendix B. Judges’ responses to adults’ productions of disyllabic tone combinations*

*Judges' Responses to adults’ disyllabic tones Productions*

| **Target tones** | **Judges' responses (%)** | | | | | | | | | | | | | | |
| --- | --- | --- | --- | --- | --- | --- | --- | --- | --- | --- | --- | --- | --- | --- | --- |
|  | **T11** | **T12** | **T13** | **T14** | **T21** | **T22** | **T23** | **T24** | **T31** | **T32** | **T34** | **T41** | **T42** | **T43** | **T44** |
| **T11** | **82** | 0 | 9 | 0 | 3 | 0 | 0 | 0 | 0 | 0 | 0 | 6 | 0 | 0 | 0 |
| **T12** | 0 | **100** | 0 | 0 | 0 | 0 | 0 | 0 | 0 | 0 | 0 | 0 | 0 | 0 | 0 |
| **T13** | 0 | 8 | **92** | 0 | 0 | 0 | 0 | 0 | 0 | 0 | 0 | 0 | 0 | 0 | 0 |
| **T14** | 0 | 0 | 3 | **94** | 0 | 0 | 0 | 3 | 0 | 0 | 0 | 0 | 0 | 0 | 0 |
| **T21** | 0 | 0 | 0 | 0 | **100** | 0 | 0 | 0 | 0 | 0 | 0 | 0 | 0 | 0 | 0 |
| **T22** | 0 | 0 | 0 | 0 | 0 | **100** | 0 | 0 | 0 | 0 | 0 | 0 | 0 | 0 | 0 |
| **T23** | 0 | 0 | 0 | 0 | 0 | 0 | **100** | 0 | 0 | 0 | 0 | 0 | 0 | 0 | 0 |
| **T24** | 0 | 0 | 0 | 0 | 0 | 0 | 0 | **100** | 0 | 0 | 0 | 0 | 0 | 0 | 0 |
| **T31** | 0 | 0 | 0 | 0 | 0 | 0 | 0 | 0 | **100** | 0 | 0 | 0 | 0 | 0 | 0 |
| **T32** | 0 | 0 | 0 | 0 | 0 | 0 | 0 | 0 | 0 | **100** | 0 | 0 | 0 | 0 | 0 |
| **T34** | 0 | 0 | 0 | 0 | 0 | 0 | 0 | 0 | 0 | 0 | **100** | 0 | 0 | 0 | 0 |
| **T41** | 0 | 3 | 0 | 0 | 0 | 0 | 0 | 0 | 3 | 0 | 0 | **94** | 0 | 0 | 0 |
| **T42** | 0 | 0 | 0 | 0 | 0 | 0 | 0 | 0 | 0 | 0 | 0 | 0 | **100** | 0 | 0 |
| **T43** | 0 | 0 | 0 | 0 | 0 | 0 | 3 | 0 | 0 | 0 | 0 | 0 | 3 | **94** | 0 |
| **T44** | 0 | 0 | 0 | 0 | 0 | 0 | 0 | 0 | 0 | 0 | 0 | 0 | 0 | 0 | **100** |

| *Judges' responses to 2- to 4-year-old children's productions of disyllabic tone combinations.* | | | | | | | | | | | | | | | |
| --- | --- | --- | --- | --- | --- | --- | --- | --- | --- | --- | --- | --- | --- | --- | --- |
| **Target tones** | **Judges' responses (%)** | | | | | | | | | | | | | | |
|  | **T11** | **T12** | **T13** | **T14** | **T21** | **T22** | **T23** | **T24** | **T31** | **T32** | **T34** | **T41** | **T42** | **T43** | **T44** |
| **T11** | **53** | 1 | 10 | 4 | 14 | 0 | 0 | 0 | 0 | 1 | 0 | 16 | 1 | 0 | 0 |
| **T12** | 2 | **45** | 20 | 1 | 1 | 0 | 9 | 0 | 0 | 10 | 0 | 2 | 6 | 0 | 3 |
| **T13** | 1 | 5 | **63** | 10 | 0 | 2 | 9 | 0 | 0 | 1 | 0 | 1 | 4 | 2 | 1 |
| **T14** | 2 | 0 | 0 | **65** | 0 | 2 | 4 | 11 | 0 | 0 | 0 | 5 | 2 | 0 | 11 |
| **T21** | 4 | 0 | 0 | 4 | **77** | 0 | 1 | 2 | 6 | 0 | 4 | 1 | 1 | 0 | 0 |
| **T22** | 0 | 6 | 7 | 2 | 3 | **49** | 19 | 1 | 0 | 3 | 0 | 5 | 4 | 0 | 0 |
| **T23** | 1 | 4 | 26 | 7 | 0 | 0 | **62** | 0 | 0 | 0 | 0 | 0 | 0 | 0 | 0 |
| **T24** | 1 | 0 | 0 | 2 | 6 | 2 | 16 | **67** | 0 | 1 | 4 | 0 | 0 | 0 | 0 |
| **T31** | 3 | 0 | 0 | 0 | 13 | 0 | 0 | 2 | **60** | 2 | 5 | 13 | 2 | 0 | 0 |
| **T32** | 0 | 1 | 1 | 3 | 1 | 1 | 0 | 1 | 2 | **80** | 4 | 3 | 1 | 0 | 1 |
| **T34** | 0 | 0 | 0 | 0 | 6 | 0 | 2 | 12 | 10 | 0 | **57** | 4 | 1 | 0 | 8 |
| **T41** | 23 | 0 | 0 | 1 | 4 | 1 | 1 | 1 | 2 | 2 | 0 | **58** | 2 | 1 | 2 |
| **T42** | 0 | 7 | 4 | 0 | 0 | 1 | 1 | 1 | 0 | 8 | 0 | 12 | **51** | 13 | 1 |
| **T43** | 0 | 2 | 13 | 0 | 0 | 0 | 0 | 0 | 0 | 2 | 0 | 0 | 16 | **63** | 3 |
| **T44** | 5 | 1 | 1 | 22 | 0 | 0 | 1 | 9 | 0 | 0 | 6 | 5 | 2 | 0 | **49** |

Note: The cells in black mark correct identification of the tones. The light shaded cells mark error patterns that constitute more than 10% of the total number of trials for the tone.
